# Basis Set Limit of CCSD(T) Energies: Explicit Correlation Versus Density-Based Basis-Set Correction

**DOI:** 10.1021/acs.jctc.3c00979

**Published:** 2023-11-11

**Authors:** Dávid Mester, Mihály Kállay

**Affiliations:** † Department of Physical Chemistry and Materials Science, Faculty of Chemical Technology and Biotechnology, Budapest University of Technology and Economics, Műegyetem rkp. 3., H-1111 Budapest, Hungary; ‡ HUN-REN-BME Quantum Chemistry Research Group, Műegyetem rkp. 3., H-1111 Budapest, Hungary; § MTA-BME Lendület Quantum Chemistry Research Group, Műegyetem rkp. 3., H-1111 Budapest, Hungary

## Abstract

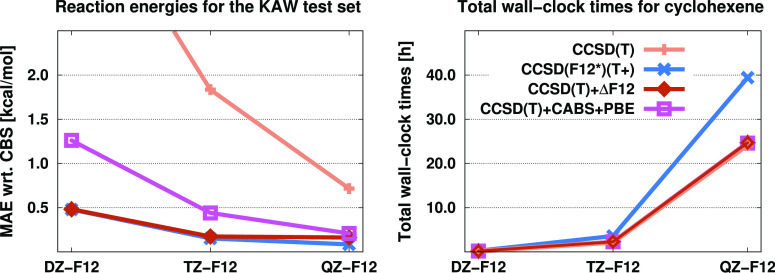

A thorough comparison is carried out for explicitly correlated and density-based basis-set correction approaches, which were primarily developed to mitigate the basis-set incompleteness error of wave function methods. An efficient implementation of the density-based scheme is also presented, utilizing the density-fitting approximation. The performance of these approaches is comprehensively tested for the second-order Møller–Plesset (MP2), coupled-cluster singles and doubles (CCSD), and CCSD with perturbative triples [CCSD(T)] methods with respect to the corresponding complete basis set references. It is demonstrated that the density-based correction together with complementary auxiliary basis set (CABS)-corrected Hartree–Fock energies is highly robust and effectively reduces the error of the standard approaches; however, it does not outperform the corresponding explicitly correlated methods. Nevertheless, what still makes the density-corrected CCSD and CCSD(T) methods competitive is that their computational costs are roughly half of those of the corresponding explicitly correlated variants. Additionally, an incremental approach for standard CCSD and CCSD(T) is introduced. In this simple scheme, the total energies are corrected with the CABS correction and explicitly correlated MP2 contributions. As demonstrated, the resulting methods yield surprisingly good results, below 1 kcal/mol for thermochemical properties even with a double-ζ basis, while their computational expenses are practically identical to those of the density-based basis-set correction approaches.

## 1 Introduction

In the realm of computational chemistry, achieving precision and reliability in electronic structure calculations is an ongoing pursuit. The main challenge lies in accounting for the electron correlation, which becomes increasingly complex as molecular systems grow in size. Two dominant approaches have emerged to tackle this issue: wave function theory (WFT) and density functional theory (DFT). Each philosophy carries its own unique strengths and limitations, shaping the landscape of modern quantum chemistry method developments.

Robust WFT-based schemes, such as the coupled-cluster (CC)^1^ and Møller–Plesset (MP)^2^ perturbation theories, offer a systematic path to accuracy through the inclusion of increasingly higher-order excitations. The CC singles and doubles (CCSD) with perturbative triples [CCSD(T)] approach,^3−5^ in particular, stands as a “gold standard” for weakly correlated systems, providing reliable results. However, the computational cost scales steeply with system size, primarily due to the slow convergence of energies and properties with the size of the one-electron basis set. This problem mainly originates from the well-known inability of WFTs to account for the electron–electron cusp of wave functions.

To address this, a possible solution is offered through explicitly correlated methods.^6−8^ By explicitly introducing interelectronic distances into the wave function, these approaches significantly enhance the basis-set convergence of the correlation energy, achieving chemical accuracy with affordable basis sets. In the case of the first explicitly correlated methods, referred to as the R12 approach,^6,9,10^ the correlation factor was linear in the interelectronic distance. The realization of this theory was first accomplished at the second-order MP (MP2) level.^10^ Significant progress occurred in the field when this correlation factor was replaced with a more sophisticated exponential factor, termed F12,^11^ which consistently provides superior results compared with the original formalism. Subsequently, numerous further developments were made over the decades, such as the fixed amplitude,^12^ density fitting (DF),^13^ and complementary auxiliary basis set (CABS)^14,15^ approaches. These advancements allowed for efficient implementations and routine applications for extended systems.^16,17^ As a result, the MP2-F12 method has become one of the most fundamental techniques in modern quantum chemistry.

Simultaneously, CC theory also reaps the benefits of the aforementioned advancements. Consequently, several groups developed and implemented explicitly correlated CCSD methods.^18−21^ Despite the outstanding accuracy of these approaches, the applicability of the rigorous CC-F12 methods was limited due to their increased computational expenses. To tackle this, several approximate methods were elaborated, efficiently reducing the complexity and cost of calculations while preserving the accuracy of the original approaches.^22−25^ Thanks to these efforts, nowadays explicitly correlated CCSD calculations can be extensively applied, even to larger systems. The treatment of higher-order excitations within the explicitly correlated formalism remains an open question to this day.^26,27^ A simple and size-consistent procedure for handling the (T) correction has been recently proposed by our group.^28^

DFT methods are widely employed in modern electronic structure calculations due to their outstanding accuracy-to-cost ratio.^29,30^ The advantages and disadvantages of DFT and WFT methods complement each other. In the DFT formalism, the central quantity is the one-body electron density instead of the complex wave function, making calculations significantly more cost-effective. Furthermore, this approach is well-suited for describing short-range interactions, which facilitates the handling of the cusp problem. In practice, this implies that the complete basis set (CBS) limit can already be achieved with smaller basis sets. However, the biggest drawback of the formalism is that the methods cannot be systematically improved. To combine the strengths of both DFT and WFT formalisms, numerous approaches have been developed over the past decades.^31,32,32−35^ One of the most promising attempts is range-separated DFT (RS-DFT).^36,37^ In this formalism, the Coulomb operator is divided into a long-range component treated with WFT and a complementary short-range part addressed using DFT. Since the WFT component effectively handles nondivergent electron–electron interactions and the semilocal functionals in DFT are good at capturing short-range interactions, this approach effectively diminishes the cusp problem and speeds up basis-set convergence.^38−51^

One of the most noteworthy approaches in recent years for reducing basis set incompleteness error (BSIE) was developed by Toulouse, Giner, and their co-workers.^52,53^ Their density-based basis-set correction relies on an RS-DFT approach in which the spatial nonhomogeneity of the BSIE is characterized by introducing a range-separation function. By utilizing this, a basis-set correction to the correlation energy can be calculated, leading to a significant improvement in the accuracy of standard WFT-based methods.^54−58^

In this article, a thorough comparison of explicitly correlated and density-based basis-set correction methods is carried out. Following a brief theoretical introduction, we present an efficient implementation of the latter by utilizing the DF approximation. Subsequently, we discuss the performance of these approaches for MP2, CCSD, and CCSD(T). To achieve this, various benchmark sets are examined with a primary focus on thermochemical properties and interaction energies. Finally, the computational requirements of the methods are assessed.

## 2 Theory

### 2.1 Explicitly Correlated Methods

In the framework of conventional MP perturbation theory,^2^ the first-order wave function is expanded as
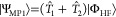
1where |Φ_HF_⟩ is the reference Hartree–Fock (HF) determinant, and  and  denote the standard single and double excitation operators, respectively, with
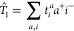
2and
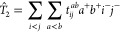
3Here, *t*_*i*_^*a*^ and *t*_*ij*_^*ab*^ stand for the first ground-state amplitudes, and indices *i*, *j*... (*a*, *b*...) refer to occupied (virtual) spin orbitals, while *p*, *q*... are used for generic molecular orbital (MO) indices. Operators *a*^+^ and *i*^–^ represent creation and annihilation operators, respectively. The MP2 correlation energy, *E*_MP2,c_, is simply obtained by substituting eq 1 into the Schrödinger equation and then projecting it onto the reference space. The resulting final expression, for canonical HF orbitals, is written as
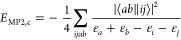
4where ⟨*ab*∥*ij*⟩ = ⟨*ab*|*ij*⟩ – ⟨*ab*|*ji*⟩ is an antisymmetrized two-electron integral using the conventional ⟨12|12⟩ notation, while ε_*p*_ denotes the corresponding orbital energy.

In explicitly correlated F12 approaches,^8,11,59^ the wave function is augmented with geminals
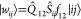
5which explicitly incorporate interelectronic distances *r*_12_ in the F12 correlation factor *f*_12_. Additionally,  is an orthogonality projector, and the rational generator  ensures the satisfaction of coalescence conditions. In practice, the functions |*w*_*ij*_⟩ are represented by an expansion in a determinant basis |αβ⟩ constructed from a formally complete virtual basis, the elements of which are labeled as α, β... In the CABS approach,^14,15,23^ this virtual basis is formed from the HF virtual MOs and a complementary MO basis. In the context of MP2-F12 theory,^16,17,60^ the first-order wave function is expanded as
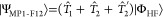
6where  generates double excitations into the second-quantized representation of the above pair functions with amplitudes *c*_*ij*_^*kl*^
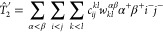
7where *w*_*kl*_^αβ^ = ⟨αβ|*w*_*kl*_⟩. Once the amplitude equations are solved, the final MP2-F12 total energy is obtained as
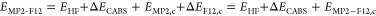
8where *E*_HF_, Δ*E*_CABS_, Δ*E*_F12,c_, and *E*_MP2–F12,c_ are the total HF energy, the CABS correction to the HF energy, the explicitly correlated contribution to the MP2 correlation energy, and the MP2-F12 correlation energy, respectively.

Explicitly correlated F12 approaches can be defined within CC theory as well. In the conventional CCSD approach,^3^ the wave function is parametrized in an exponential form as
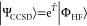
9where *T̂* is the cluster operator defined as . Using this expression, the CCSD correlation energy and the corresponding cluster amplitude equations are read as
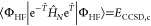
10
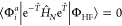
11and
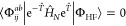
12where  represents the normal-ordered Hamiltonian, and ⟨Φ_*i*_^*a*^| and ⟨Φ_*ij*_^*ab*^| are singly and doubly excited determinants, respectively. In explicitly correlated CCSD approaches,^19,21,23,25,61^ akin to the MP2 method, the cluster operator incorporates the additional  operator. While the aforementioned equations remain applicable in this explicitly correlated context with the modified cluster operator, an additional set of equations
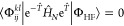
13is required to determine the *c*_*ij*_^*kl*^ coefficients.

This explicitly correlated CCSD approach can be further enhanced through the incorporation of (T) corrections.^26−28^ However, this augmentation is not straightforward. In our recent paper,^28^ a size-consistent perturbative triples correction, termed (T+), has been proposed. In this scheme, the MP2 and MP2-F12 correlation energies, along with the triples correction, are decomposed into individual contributions from occupied MOs, respectively, as *E*_MP2,c_ = ∑_*i*_δ*E*_*i*_^MP2^, *E*_MP2-F12,c_ = ∑_*i*_δ*E*_*i*_^MP2-F12^, and *E*_(T)_ = ∑_*i*_δ*E*_*i*_^(T)^. Thereafter, the contribution of each MO to the (T) correction is separately scaled with the ratio of the corresponding increments δ*E*_*i*_^MP2-F12^ and δ*E*_*i*_^MP2^ as
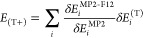
14

### 2.2 Density-Based Basis-Set Correction

By applying density-based basis-set correction to the energy calculated in a given one-electron basis set, , of a specific method, the CBS value of the method can be approximated.^52,53^ A suitable basis-dependent complementary density functional, , with *n* as the electron density, has been proposed by Giner, Toulouse, and their co-workers,^53^ and its effectiveness has been demonstrated for the “gold-standard” CCSD(T) approach using the following formula
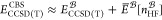
15where *E*_CCSD(T)_^CBS^ and  are the CCSD(T) energies with a CBS and basis set , respectively, and  refers to the HF density evaluated with . The main objective of this correction is to account for the missing part of short-range correlation effects arising due to the incompleteness of the one-electron basis set. Consequently, the energy correction is approximated using a short-range density functional that complements a nondivergent long-range interaction properly described by WFT.

The coupling of DFT and WFT is achieved through the definition of a real-space representation for the electron–electron Coulomb operator projected onto the selected basis set. This general effective two-electron interaction operator is defined^52^ as
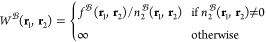
16where
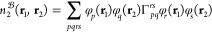
17 is a general opposite-spin pair density, ϕ_*p*_(**r**_1_) stands for an MO, and
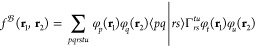
18As demonstrated in ref (52), this effective operator satisfies the  condition. Upon forming the effective operator, it is linked to a local range-separation function that automatically adapts to quantify the incompleteness of  in the spatial domain. For this function, the following formula has been suggested^53^
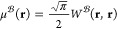
19Once μ(**r**) is evaluated, the complementary energy correction is approximated using a Perdew–Burke–Ernzerhof (PBE)-based correlation functional^53,62,63^ as
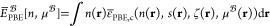
20where ζ is the spin polarization, and *s* is the reduced density gradient, while  interpolates between the standard PBE correlation functional,^64^ ε_PBE,c_(*n*, *s*, ζ), at μ = 0 and the exact large-μ behavior^39,65,66^ yielding
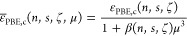
21with
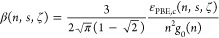
22where *g*_0_(*n*) represents the uniform electron gas on-top pair-distribution function.^66,67^

The rate-determining step of the above procedure is the construction of the effective operator , particularly the expression presented in eq 18. Nonetheless, various approximations can be introduced to accelerate the calculations. For instance, as demonstrated in refs (52) and (53), for weakly correlated systems, the application of the HF pair density is well-justified. In this case,  can be calculated as
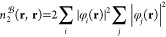
23while  reads as
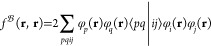
24The evaluation of this contribution to  scales as *N*_grid_*N*_occ_^2^*N*_basis_^2^, where *N*_grid_, *N*_occ_, and *N*_basis_ are the number of grid points, the number of occupied MOs, and the total number of MOs in the basis set , respectively.

In addition, as we demonstrate here, the computational expenses and memory requirements can be further decreased by utilizing the DF approximation. In this approach, the four-center, two-electron integrals can be recast as
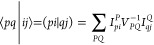
25where *P* and *Q* stand for the elements of an auxiliary basis, whereas *I*_*pi*_^*P*^ and *V*_*PQ*_ are three- and two-center Coulomb integrals, respectively, and *V*_*PQ*_^–1^ is a simplified notation for the corresponding element of the inverse of the two-center Coulomb integral matrix. Usually, the matrix **K** with elements *K*_*pi*,*qj*_ = (*pi*|*qj*) is factorized as **K** = **IV**^–1/2^**V**^–1/2^**I**^T^ = **JJ**^T^. Using the latter notation, the intermediate  can be expressed as
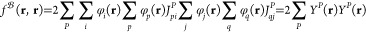
26with
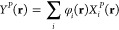
27and
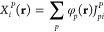
28Here, the most expensive step is the contraction of the three-center integrals **J** and the real-space representation of the MOs, eq 28, and its complexity scales only as *N*_grid_*N*_occ_*N*_basis_*N*_aux_, where *N*_aux_ stands for the number of auxiliary functions. That is, the fifth-power scaling of the density-based correction can be reduced to the fourth-power scaling using our algorithm. Assuming that *N*_aux_ is roughly three times larger than *N*_basis_, the ratio of the CPU requirements of the conventional and DF algorithms is proportional to *N*_occ_/3. Accordingly, the benefits become more pronounced with increasing system sizes than with increasing the quality of the basis set. In addition, storing the above three-index arrays in the main memory is conveniently feasible, while the size of the two-electron integrals rapidly becomes cumbersome beyond a certain size range. Consequently, the size of the studied systems can be further increased when the DF approximation is invoked.

Although the density-based basis-set correction was also developed to cure the incompleteness of the one-electron basis set, it has not yet been thoroughly compared to the F12-based methods. Furthermore, we also explore additional advancements concerning the complementary energy correction. Since the selection of the PBE correlation functional is arbitrary in eq 23, we further investigate the application of other popular correlation functionals.

### 2.3 MP2-Based Basis-Set Correction

Motivated by the similar runtimes required for the MP2-F12 calculations and the evaluation of the density-based correction (see Section 4.4), here, we propose a very simple incremental approach based upon MP2 to reduce the BSIE of conventional CCSD and CCSD(T). In the case of CCSD, the corrected CCSD total energy, hereafter denoted by CCSD + ΔF12, is calculated as
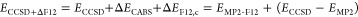
29where *E*_CCSD_ and *E*_MP2_ are the total CCSD and MP2 energy, respectively, which can be interpreted in two ways. On the one hand, the obtained energy is the standard CCSD energy corrected with the HF CABS correction and the F12 correlation contribution obtained from an MP2-F12 calculation (cf. eq 8). On the other hand, it can also be regarded as the MP2-F12 energy corrected with the difference between conventional CCSD and MP2 energies. Anyway, the CCSD + ΔF12 energy converges to the CBS limit CCSD energy with an increasing cardinal number of the basis set.

The MP2-corrected CCSD(T) energy, CCSD(T) + ΔF12, can be analogously defined by replacing the CCSD energy with the CCSD(T) energy in the above equation. Alternatively, instead of (T), the (T+) correction can also be evaluated, which also improves the basis set convergence of the perturbative triples contributions. Note that the necessary scaling factors can be simply computed as a byproduct of the MP2-F12 calculation.

It is also pertinent to comment on the scaling of the ΔF12 correction. Its computational costs are identical with those of an MP2-F12 calculation. If the DF approximation is employed, the latter are dominated by the assembly of the four-center integrals of the *f*_12_ correlation factor from the corresponding three-center ones. This scales as , where *N*_basis′_ is the size of the joint HF MO plus complementary MO basis. Since the evaluation of the ΔF12 correction scales as the fifth power of the system size, the density-based correction may be more advantageous beyond a particular system size. Nonetheless, *N*_grid_, which contributes to the scaling of the density-based correction, can also be very large, resulting in a considerable prefactor. Anyway, as we will see, the wall-clock times are massively determined by the standard CC calculations.

## 3 Computational Details

The density-based basis-set correction, relying on ref (53), has been implemented in the MRCC suite of quantum chemical programs.^68,69^ The technical details of our explicitly correlated CCSD(T) implementation have been discussed in refs (28) and (70). For the explicitly correlated MP2 and CCSD calculations, the MP2-F12^17^ and CCSD(F12*)^25^ approaches were utilized, respectively, supposing ansatz 2B, the F + K commutator approximation, and fixed amplitudes.^12,17,71^ Restricted open-shell HF references with semicanonical orbitals were used for the open-shell systems.^72^ The frozen core approximation was applied in all post-HF calculations.

The performances of the approaches were extensively tested for various basis sets. Accordingly, as the atomic orbital (AO) basis set, the correlation consistent cc-pV*X*Z-F12 (X = D, T, and Q)^73^ and aug-cc-pV*X*Z (X = D, T, Q, 5, and 6)^74−78^ basis sets were employed. For the sake of brevity, the cc-pV*X*Z-F12 and aug-cc-pV*X*Z basis sets will be referred to as *X*Z-F12 and a*X*Z, respectively. The DF approximation was invoked at both the HF and post-HF levels. In the standard calculations, the corresponding fitting bases of Weigend^79,80^ were applied, while the aug-cc-pV(*X* + 1)Z-RI-JK and the aug-cc-pwCV(*X* + 1)Z-RI bases of Hättig^81^ were used for the explicitly correlated calculations. For the CABS, the corresponding “OPTRI” bases of Yousaf and Peterson^82,83^ were applied.

For the complementary energy correction, the PBE,^64^ Perdew 1986 (P86),^84^ and Perdew–Wang 1992 (PW91)^85^ correlation functionals were tested. The corresponding functionals were obtained from the Libxc library.^86,87^ The default adaptive integration grid of the MRCC package was used for the correlation contributions, while the tolerance for the accuracy of angular integration was set to 10^–10^ and 10^–11^ a.u. for the *X*Z-F12 and a*X*Z basis sets, respectively. The tighter value was necessary because numerical instability was observed for the P86 functional in the case of diffuse functions.

For benchmarking the methods for thermochemistry, the test set of Knizia, Adler, and Werner (KAW)^27^ was used. This set includes 49, 28, and 48 atomization energies and reaction energies of closed- and open-shell systems, respectively, involving 66 species. The reference CBS values were taken from two-point extrapolated a(5,6)Z energies.^28,88^ The performance for interaction energies was benchmarked for the A24 test^89^ of Řezáč and Hobza. This compilation contains 24 complexes of small molecules bound by noncovalent interactions. The reference CBS values were calculated from two-point extrapolated a(4,5)Z energies. In order to make a comprehensive comparison, both counterpoise (CP)-uncorrected and -corrected values are discussed for the results obtained with double- and triple-ζ basis sets. The wall-clock time measurements for the cyclohexene molecule^90^ were carried out on an 8-core Intel Xeon E5–2609 v4 processor running at 1.7 GHz.

The primary statistical error measure presented in the figures and tables is the mean absolute error (MAE). The Supporting Information includes additional metrics such as the root-mean-square error and maximum absolute error. The chemical properties discussed above were calculated from the total energies. In Supporting Information, the detailed results are available for the correlation energies as well.

## 4 Results and Discussion

### 4.1 Atomization Energies

**Figure 1:**
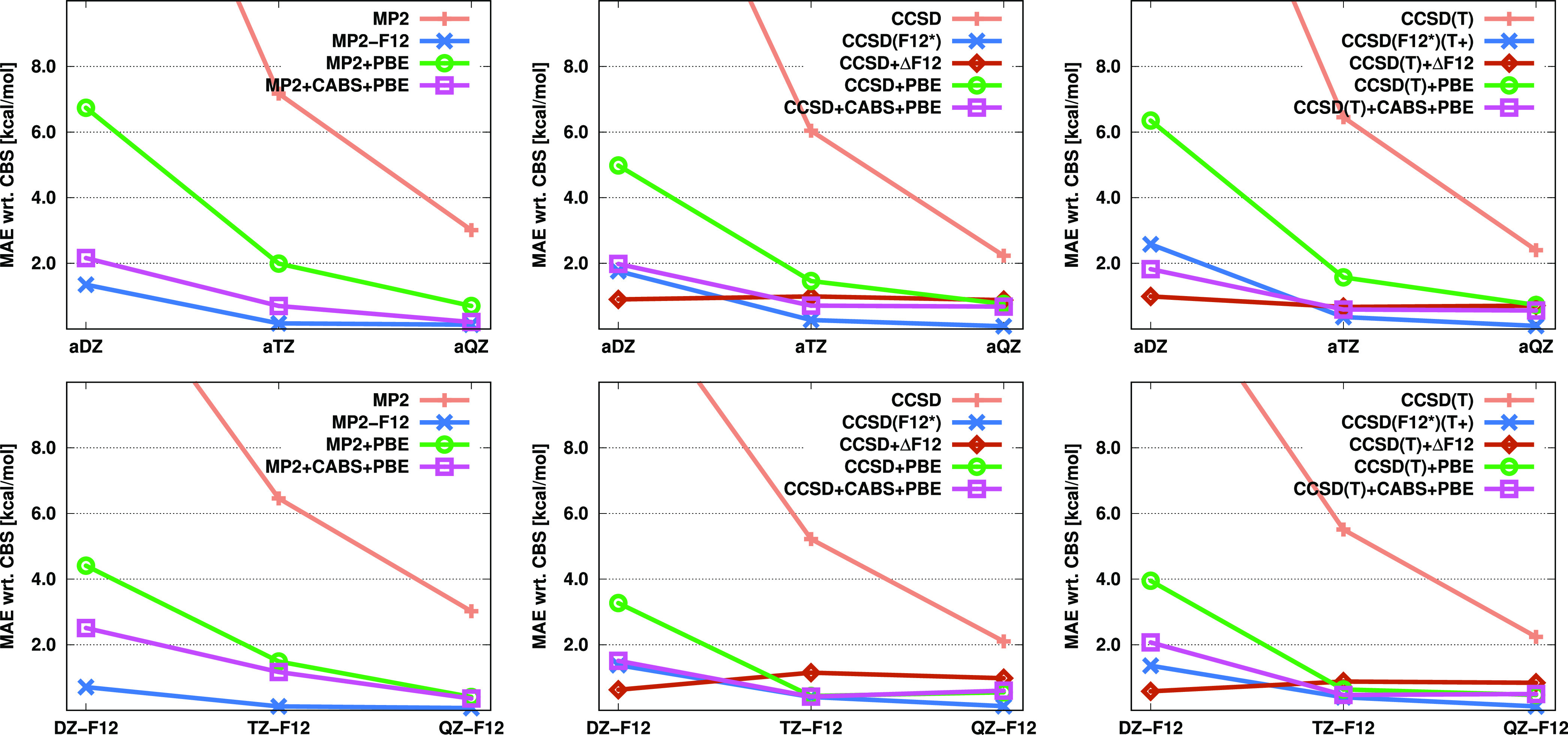
MAEs (in kcal/mol) for atomization energies of the KAW test set^27^ for the standard, F12, and corrected MP2, CCSD, and CCSD(T) methods using various basis sets.

To assess the performances, we first discuss the atomization energies for the KAW test set.^27^ The numerical results are listed in Figure 1. Inspecting the MP2-based results, it can be concluded that the best performance is achieved by MP2-F12, regardless of the considered basis set. For this approach, the MAE already drops below 0.2 kcal/mol, even with a triple-ζ basis. As can be seen, the CABS correction significantly enhances the performance of the density-based correction, especially with smaller basis sets, and the improvements are more pronounced with the a*X*Z basis sets. The error decreases below 1 kcal/mol for MP2 + CABS + PBE using the aTZ basis sets, while it is 1.17 and 0.36 kcal/mol with the TZ-F12 and QZ-F12 basis sets, respectively. That is, a slightly slower convergence is observed with the *X*Z-F12 basis sets.

In regard to the CCSD-based methods, inspecting the errors obtained with double-ζ basis sets, the best results are attained by CCSD + ΔF12. In this case, the MAEs are only 0.90 and 0.63 kcal/mol using the aDZ and DZ-F12 basis sets, respectively, although the errors do not rigorously decrease with increasing cardinal number. The performance of CCSD + CABS + PBE is highly similar to CCSD(F12*). With a triple-ζ basis, the MAEs for both approaches drop below 1 kcal/mol; however, a somewhat better convergence is realized using a quadruple-ζ basis for the latter method, especially with diffuse functions.

Similar considerations can be made if the CCSD(T) results are assessed. Again, the performance of the CCSD(T)+ΔF12 method is quite surprising with MAEs below 1 kcal/mol, regardless of the basis set used. The results are somewhat less sensitive using the a*X*Z basis sets. Comparing the CCSD(F12*)(T+) and CCSD(T) + CABS + PBE methods, it can be stated that the former provides slightly more accurate results with aDZ basis sets, while the latter performs slightly better when DZ-F12 basis sets are used. The difference decreases when a triple-ζ basis is applied, resulting in MAEs around 0.4 kcal/mol in both cases; however, the errors do not decrease with the quadruple-ζ basis for the density-corrected method.

The dependence of the performance of the density-based correction on the correlation functional employed is also analyzed. Since the application of the CABS correction is clearly advantageous, we will discuss only the results obtained with the latter. The corresponding MAEs are collected in Table 1. The results are presented in comparison to PBE, which was chosen in the original paper of Giner, Toulouse et al.^53^ As can be seen, the performance of the PBE and PW91 functionals is practically identical. The largest difference for the entire set is only 0.01 kcal/mol. In the case of P86, larger deviations are observable; however, they are not consistent. With the aDZ basis set, the errors are slightly larger for all methods, while when a larger basis is used, the difference decreases. On the other hand, with the *X*Z-F12 basis sets, the errors are somewhat smaller, except for the MP2/QZ-F12 level. However, since the difference does not exceed 0.1 kcal/mol in either case, the deviation cannot be considered significant.

**Table 1:** MAEs (in kcal/mol) for Atomization Energies of the KAW Test Set^27^ Using Various Basis Sets and Correlation Functionals

	MP2			CCSD			CCSD(T)		
basis set	PBE + CABS	P86 + CABS	PW91 + CABS	PBE + CABS	P86 + CABS	PW91 + CABS	PBE + CABS	P86 + CABS	PW91 + CABS
aDZ	2.16	2.19	2.16	1.98	2.04	1.97	1.82	1.90	1.83
aTZ	0.70	0.69	0.70	0.71	0.70	0.70	0.59	0.58	0.59
aQZ	0.21	0.22	0.21	0.68	0.67	0.68	0.56	0.55	0.55
DZ-F12	2.51	2.42	2.51	1.51	1.48	1.52	2.07	1.98	2.07
TZ-F12	1.17	1.15	1.17	0.42	0.43	0.42	0.47	0.47	0.48
QZ-F12	0.36	0.45	0.36	0.60	0.52	0.60	0.50	0.45	0.50

### 4.2 Closed- and Open-Shell Reaction Energies

**Figure 2:**
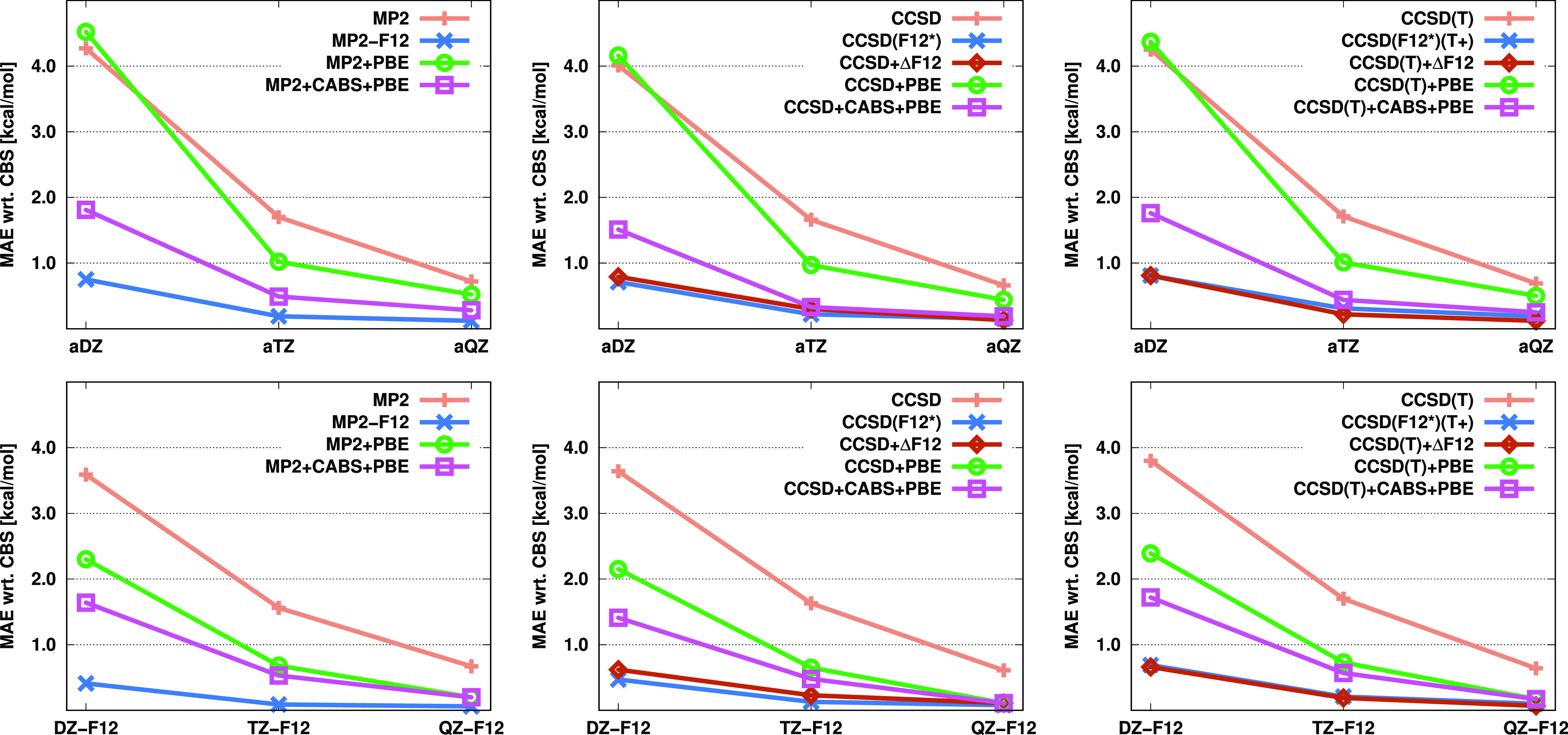
MAEs (in kcal/mol) for closed-shell reaction energies of the KAW test set^27^ for the standard, F12, and corrected MP2, CCSD, and CCSD(T) methods using various basis sets.

Next, we analyze the errors of the closed-shell reaction energies obtained for the KAW test set.^27^ The results are listed in Figure 2. In the case of the MP2-based results, the outcomes closely resemble those observed for the atomization energies. Similarly, competing with the performance of MP2-F12 is challenging. The MAEs are already below 1 kcal/mol even with a double-ζ basis, and the errors decrease further with an increasing cardinal number. The CABS correction still significantly enhances the performance of the density-based corrections, and the improvement is more pronounced with the a*X*Z basis sets. For MP2 + CABS + PBE, the application of a triple-ζ basis already provides satisfactory results with MAEs of 0.49 and 0.53 kcal/mol using the augmented and F12 basis sets, respectively.

The CCSD and CCSD(T)-based results can be discussed together. It can be observed that the performance of the original F12-based methods and our incremental approach are practically identical. The largest difference between the two methods is approximately 0.1 kcal/mol. The errors are already below 1 kcal/mol with a double-ζ basis, and the MAEs monotonically decrease with increasing cardinal number. An almost complete convergence can be achieved with a triple-ζ basis and MAEs of around 0.2 kcal/mol. The CABS-corrected CC + PBE errors with a double-ζ basis range between 1.5 and 1.8 kcal/mol, while they are around 0.5 kcal/mol using a triple-ζ basis. In addition, if augmented basis sets are used, the accuracy is closer to that provided by more accurate WFT-based methods.

The results regarding the correlation functionals are summarized in Table 2. It can be seen that, similar to the previous results, the PBE and PW91 functionals provide practically identical MAEs. The largest deviation still does not exceed 0.01 kcal/mol. Outlier values are produced only by P86. In contrast to the results obtained for atomization energies, in comparison with the other functionals, the MAEs are smaller for P86 with the a*X*Z basis sets, while they are somewhat larger using the *X*Z-F12 basis sets. However, the errors are still not significant, and the differences between them decrease with increasing cardinal numbers. With a triple-ζ basis, which is the most relevant from a practical perspective, the difference is less than 0.03 kcal/mol.

**Table 2:** MAEs (in kcal/mol) for Closed-Shell Reaction Energies of the KAW Test Set^27^ Using Various Basis Sets and Correlation Functionals

	MP2			CCSD			CCSD(T)		
basis set	PBE + CABS	P86 + CABS	PW91 + CABS	PBE + CABS	P86 + CABS	PW91 + CABS	PBE + CABS	P86 + CABS	PW91 + CABS
aDZ	1.81	1.44	1.80	1.51	1.38	1.51	1.76	1.46	1.76
aTZ	0.49	0.48	0.49	0.33	0.36	0.33	0.44	0.43	0.43
aQZ	0.28	0.27	0.27	0.19	0.18	0.18	0.25	0.25	0.25
DZ-F12	1.64	1.82	1.65	1.41	1.56	1.42	1.72	1.88	1.72
TZ-F12	0.53	0.53	0.53	0.48	0.48	0.48	0.57	0.57	0.57
QZ-F12	0.20	0.21	0.20	0.11	0.13	0.11	0.17	0.18	0.17

**Figure 3:**
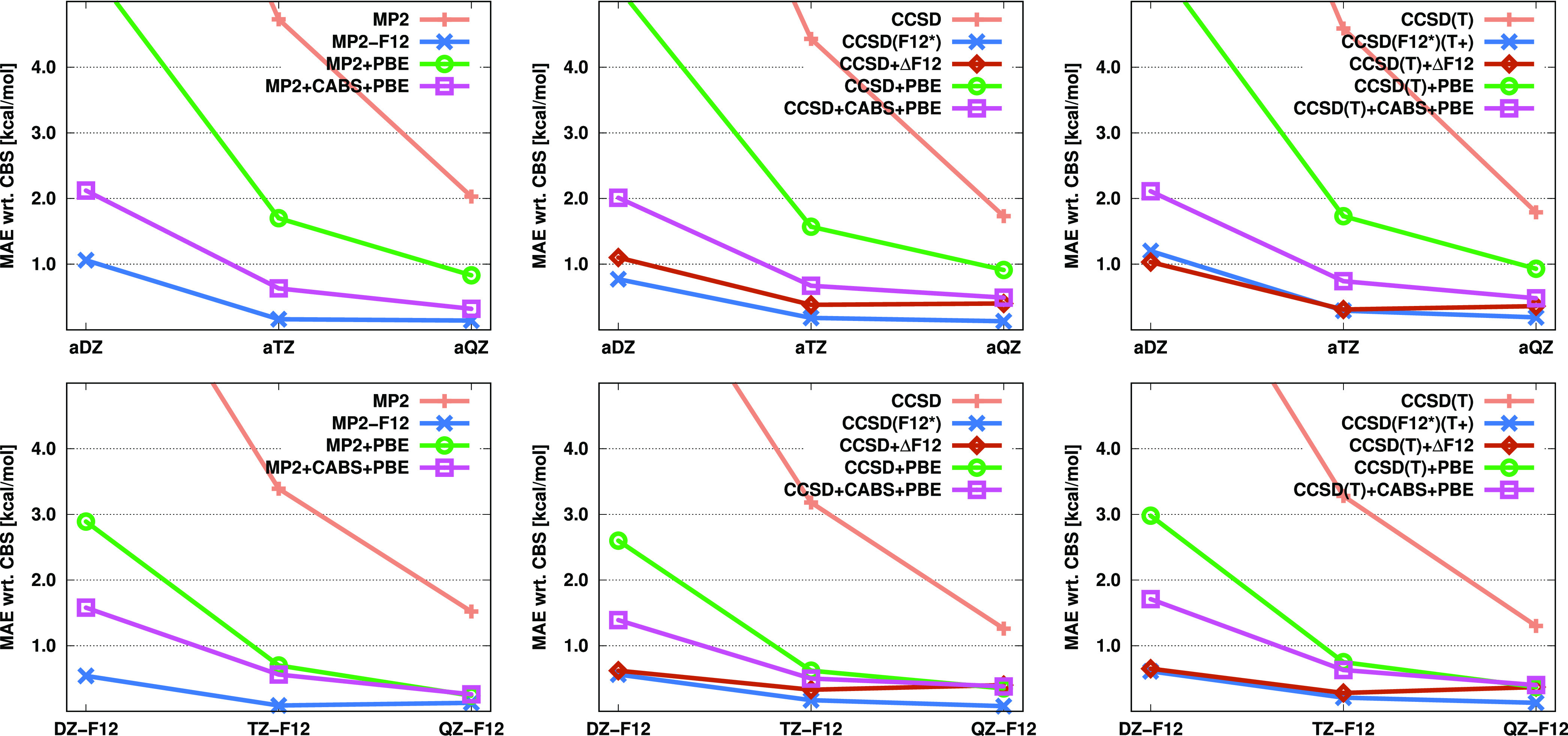
MAEs (in kcal/mol) for open-shell reaction energies of the KAW test set^27^ for the standard, F12, and corrected MP2, CCSD, and CCSD(T) methods using various basis sets.

The errors obtained for open-shell reaction energies are listed in Figure 3. In general, we can conclude that, for the standard methods, the errors are much more significant compared to the values obtained for the closed-shell reactions. For each standard WFT-based method, the MAE exceeds 10 kcal/mol with a double-ζ basis. In contrast, for the CABS-corrected CC + PBE approach, the errors are approximately 2.0 and 1.5 kcal/mol with the aDZ and DZ-F12 basis sets, respectively. In the case of MP2, the MP2-F12 approach is still the method of choice, where BSIE is practically eliminated with a triple-ζ basis. For CCSD and CCSD(T), the performance of the incremental methods is excellent, and the MAEs are consistent with those provided by the original F12 approaches even with smaller basis sets. For the CABS-corrected CC + PBE approach, the errors drop below 1 kcal/mol using a triple-ζ basis; however, our incremental scheme still provides more reliable results.

Regarding the correlation functionals, the results are compiled in Table 3. As can be seen, highly similar conclusions can be drawn as for the closed-shell reaction energies. Despite the identical performances of PBE and PW91, P86 may yield outlier values with smaller basis sets. The former functionals remain consistently more accurate using *X*Z-F12 basis sets. For MP2, a somewhat smaller MAE is attained by P86 employing aDZ basis sets; however, in contrast to the previous cases, the PBE and PW91 functionals provide better results for CCSD and CCSD(T) using the same basis sets. Needless to say, the difference between the errors decreases with an increasing cardinal number in all cases.

**Table 3:** MAEs (in kcal/mol) for Open-Shell Reaction Energies of the KAW Test Set^27^ Using Various Basis Sets and Correlation Functionals

	MP2			CCSD			CCSD(T)		
basis set	PBE + CABS	P86 + CABS	PW91 + CABS	PBE + CABS	P86 + CABS	PW91 + CABS	PBE + CABS	P86 + CABS	PW91 + CABS
aDZ	2.12	2.06	2.12	2.01	2.16	2.02	2.11	2.12	2.12
aTZ	0.63	0.63	0.63	0.67	0.64	0.67	0.74	0.74	0.74
aQZ	0.32	0.34	0.32	0.49	0.50	0.49	0.48	0.49	0.48
DZ-F12	1.58	1.82	1.59	1.39	1.64	1.40	1.71	1.96	1.72
TZ-F12	0.56	0.56	0.57	0.50	0.51	0.50	0.63	0.63	0.63
QZ-F12	0.26	0.28	0.26	0.38	0.36	0.38	0.40	0.38	0.40

### 4.3 Interaction Energies

**Figure 4:**
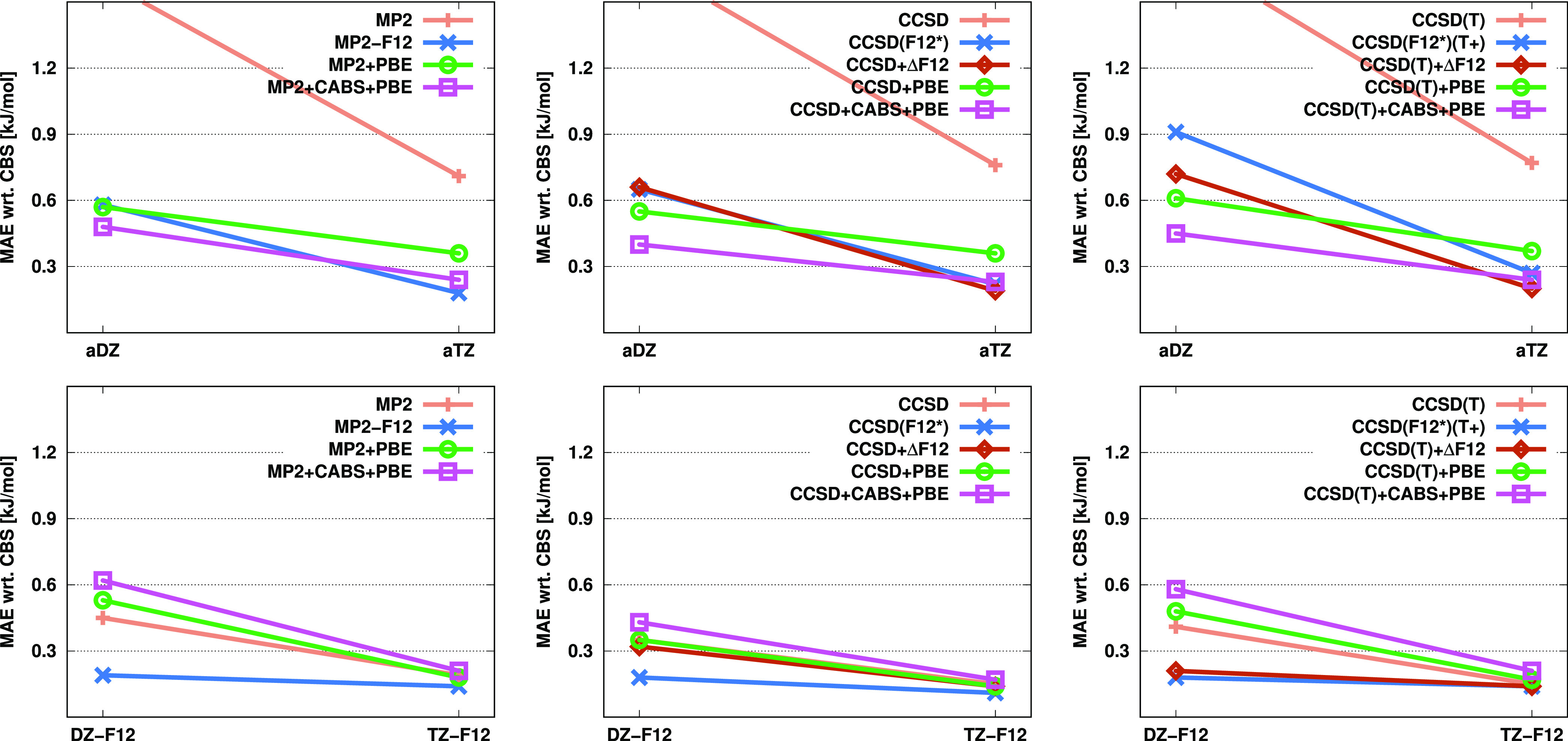
MAEs (in kJ/mol) for CP-uncorrected interaction energies of the A24 test set^89^ for the standard, F12, and corrected MP2, CCSD, and CCSD(T) methods using various basis sets.

Finally, we benchmark interaction energies on the A24 test set.^89^ First, the CP-uncorrected results are discussed and visualized in Figure 4. Analyzing them, we can conclude that quite distinct trends can be observed with the a*X*Z and *X*Z-F12 basis sets. In the former case, the largest errors are consistently obtained by standard methods. The MAE is 1.6 kJ/mol for the standard MP2 approach using aDZ basis sets, whereas this value is between 0.5 and 0.6 kJ/mol for MP2-F12 and the density-based corrected methods with the same basis sets and decreases roughly by half for all of the approaches when the aTZ basis is applied. Similar results are observed for CCSD as well. Our incremental method is in perfect agreement with CCSD(F12*). The lowest MAE using the aDZ basis set is provided by CCSD + CABS + PBE, while with larger basis sets, the incremental approach performs slightly better. However, significant differences in performance among these methods cannot be noted. The CCSD(T) results show somewhat higher deviations. The accuracy of the standard method remains unchanged, while the MAEs of the remaining approaches range between 0.90 and 0.45 kJ/mol using the aDZ basis sets. The higher value corresponds to the original F12 method, while the lower one corresponds to the CABS-corrected PBE approach. Nevertheless, when the larger basis set is applied, the incremental method becomes the most accurate by a small margin. In general, it can also be concluded that the CABS correction does not play such a crucial role for interaction energies in conjunction with the a*X*Z basis sets.

With the *X*Z-F12 basis sets, the outcomes change somewhat. Most notably, the errors significantly decrease, especially for CCSD and CCSD(T), with these basis sets including less diffuse functions. For MP2, the best results are still provided by MP2-F12, with MAEs of 0.19 and 0.14 kJ/mol using the DZ-F12 and TZ-F12 basis sets, respectively. Surprisingly, the standard MP2 method is more accurate with the smaller basis sets than the density-corrected approaches; however, the error for the latter methods also drops to around 0.2 kJ/mol when applying the TZ-F12 basis sets. For CCSD, the deviations are slightly smaller. The best result is still attained by the standard F12 approach. Next is our incremental method, where the MAE decreases from 0.32 to 0.14 kJ/mol with increasing cardinal number, while the performance of the PBE-based approaches is quite similar. For CCSD(T), the original F12 method and the incremental method practically yield identical results. The MAEs are already around 0.2 kJ/mol with the double-ζ basis, decreasing further to 0.14 kJ/mol using the TZ-F12 basis sets. For the remaining approaches, the errors are significantly larger with the smaller basis sets, ranging between 0.4 and 0.6 kJ/mol for the standard CCSD(T) and CCSD(T) + CABS + PBE methods, respectively. The difference among methods diminishes using a triple-ζ basis. Interestingly, the CABS correction somewhat worsens the results with these basis sets. For the comparison of the correlation functionals, the results are collected in Table 4. Inspecting the MAEs, it is demonstrated that there is no advantage to applying the P86 correlation functional in this case. The performance of PBE and PW91 remains consistent, while systematically larger errors are obtained by P86. The differences diminish with increasing cardinal number for all methods, and the MAEs are practically identical with the triple-ζ basis sets; however, there is no reason to use the P86 functional.

**Table 4:** MAEs (in kJ/mol) for Interaction Energies of the A24 Test Set^89^ Using Various Basis Sets and Correlation Functionals

	MP2			CCSD			CCSD(T)		
basis set	PBE + CABS	P86 + CABS	PW91 + CABS	PBE + CABS	P86 + CABS	PW91 + CABS	PBE + CABS	P86 + CABS	PW91 + CABS
aDZ	0.48	0.53	0.49	0.40	0.45	0.41	0.45	0.50	0.45
aTZ	0.24	0.25	0.24	0.23	0.23	0.22	0.24	0.24	0.24
DZ-F12	0.62	0.78	0.63	0.43	0.67	0.44	0.58	0.75	0.59
TZ-F12	0.21	0.22	0.22	0.17	0.18	0.17	0.21	0.22	0.21

**Figure 5:**
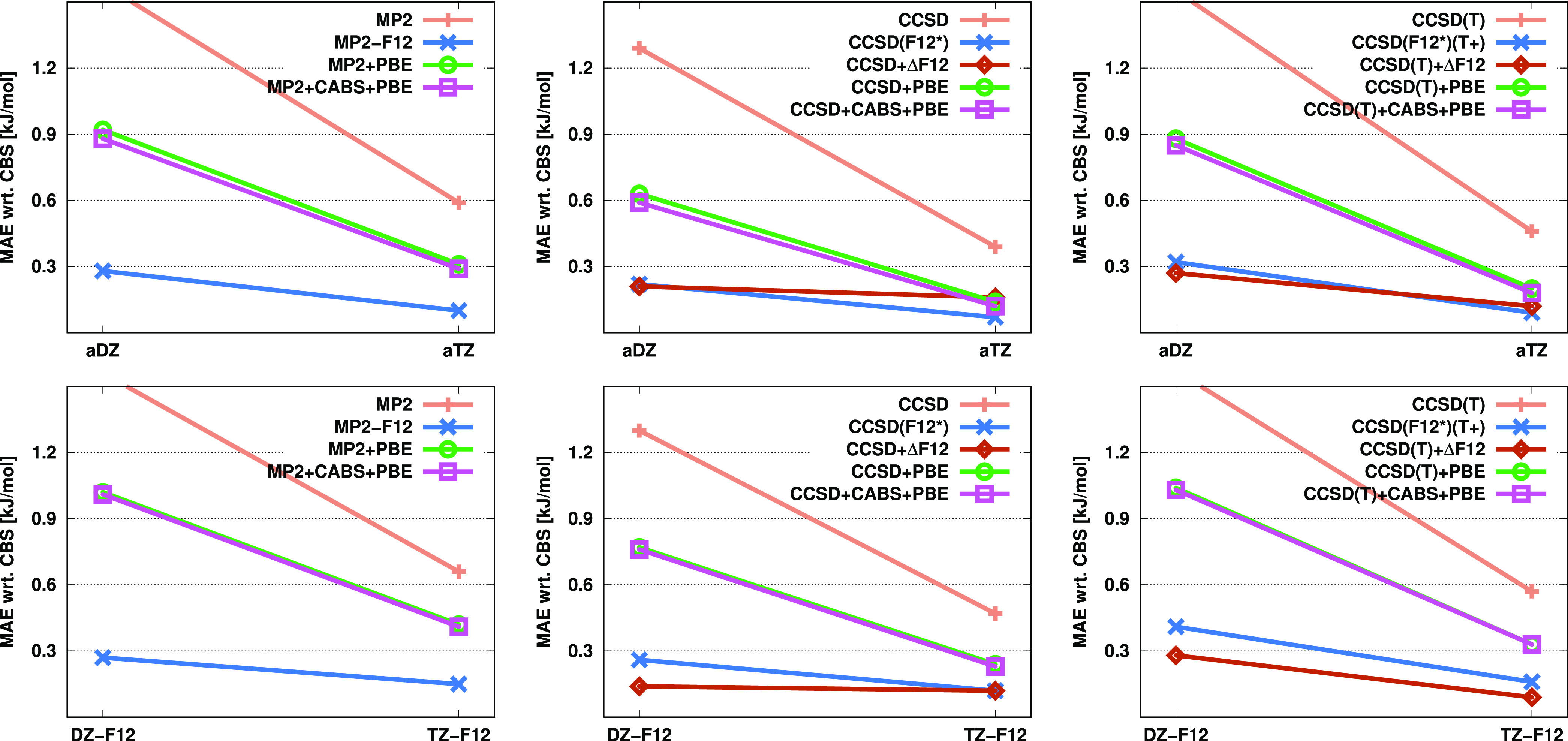
MAEs (in kJ/mol) for CP-corrected interaction energies of the A24 test set^89^ for the standard, F12, and corrected MP2, CCSD, and CCSD(T) methods using various basis sets.

The numerical results for the CP-corrected interaction energies are listed in Figure 5. In this case, it is advisable to compare the methods among themselves and to also assess the CP-corrected values alongside the uncorrected results. For MP2 using a double-ζ basis, there is a significant increase in the error for both standard and PBE-based methods. In contrast, the error notably decreases for MP2-F12 with the a*X*Z basis sets, while it remains practically unchanged using the *X*Z-F12 basis sets. Similar findings can be made regarding the triple-ζ basis sets, although the differences are somewhat smaller, of course. Therefore, based on the CP-corrected energies, the F12 approach is clearly the method of choice. Similar trends can be observed for CCSD and CCSD(T) as well. With the a*X*Z basis sets, the performance of standard and PBE-based approaches slightly deteriorates, whereas the results of F12 and our incremental methods improve significantly. In contrast, with the *X*Z-F12 basis sets, the performance of the former approaches worsens significantly, while the errors with the latter methods remain practically unchanged. Consequently, the approaches exhibit more pronounced differences, and the F12 and the incremental methods clearly outperform the others. Additionally, it is worth noting that, in this case, the CABS correction does not affect the results for the PBE-based methods. Comparing the correlation functionals, it can be concluded that the order of the functionals remains unchanged. The PBE and PW91 values are identical, while P86 is still considered less preferable. To maintain the compactness of the manuscript, these results will not be discussed in detail.

### 4.4 Timings

The performance of the methods in terms of accuracy is discussed in detail in the previous sections. However, from the perspective of applications, the cost of the approaches is also important. In order to address this concisely in a brief study, we measured the computation times required for the methods. These benchmark calculations were carried out on the cyclohexene molecule from ref (90) using *X*Z-F12 basis sets. The DZ-F12, TZ-F12, and QZ-F12 basis sets comprise 270, 498, and 862 AOs, respectively, for this system. The wall-clock times required for the corresponding steps are compiled in Table 5. Comparing the individual steps, it can be generally stated that the computation times necessary for MP2-F12 are roughly identical to those for the density-based basis-set correction, and the evaluation of the CABS correction requires moderate resources. In other words, MP2-F12 and MP2 + CABS + PBE calculations have similar requirements. Furthermore, it can be observed that the CCSD(F12*) method is approximately twice as expensive as the standard CCSD approach and that there is no significant difference in the computation of the (T) and (T+) terms. Based on these findings, it can be concluded that our incremental method has a cost similar to the density-based basis-set correction scheme, and these calculations can be performed at roughly half of the price compared to the corresponding CCSD(F12*) or CCSD(F12*)(T+) calculation. Concerning the precise values, using the TZ-F12 basis set, the CCSD(F12*)(T+) calculation takes about 3.5 h, while the CCSD(T) + CABS + PBE and CCSD(T) + ΔF12 calculations require around 2.3 h. Similarly, using the QZ-F12 (DZ-F12) basis set, these wall-clock times are approximately 39.5 (0.27) and 24.6 (0.18) h for the F12 and corrected approaches, respectively. Furthermore, the corrections increase the costs of standard calculations by only a few percent, which, considering the performance in terms of accuracy, represents a reasonable and affordable price.

**Table 5:** Wall-Clock Times (in Minutes) Required for the Corresponding Steps for the Cyclohexene Molecule Using Various Basis Sets

	F12^a^			standard			correction	
basis set	MP2-F12	CCSD(F12*)	(T+)	MP2	CCSD	(T)	density-based	CABS
DZ-F12	1.1	9.9	5.1	0.1	4.7	4.9	1.4	0.2
TZ-F12	5.5	148.7	58.1	0.3	71.8	57.9	7.8	0.7
QZ-F12	27.6	1826.9	508.2	1.4	947.4	501.3	24.1	1.6

a Excluding the computation time of the CABS correction.

## 5 Conclusions

In this study, a detailed comparison was carried out for explicitly correlated and density-based basis-set-corrected WFT methods, which were primarily developed to reduce the BSIE. Efficient implementations of the former approaches were previously established, while the latter schemes represent relatively novel advancements in the field. Herein, we first presented an efficient implementation for the density-based correction, where the density-fitting approximation was utilized, resulting in a cost-effective procedure that scales as the fourth power of the system size. The flexible framework also enables the application of arbitrary correlation functionals in the correction calculation.

Subsequently, the performance of the methods was thoroughly tested. We investigated how individual approaches mitigate basis-set errors with respect to the CBS limit. To this end, thermochemical properties, such as atomization, closed- and open-shell reaction energies, and interaction energies, were studied. Regarding the density-based basis-set correction, we can conclude that the CABS correction is highly beneficial for thermochemistry. In this case, the correction consistently reduces basis set errors, particularly with small basis sets, and exhibits greater enhancement if a*X*Z basis sets are used. When comparing the performances of the a*X*Z and *X*Z-F12 basis sets, no significant difference is observed for the CABS-corrected schemes. Furthermore, the approach equally reduces the errors for MP2, CCSD, and CCSD(T). Hence, the robustness of the method is confirmed.

Regarding accuracy, it is clear that the approach does not outperform the corresponding CCSD(F12*) and CCSD(F12*)(T+) methods. Nevertheless, it still remains competitive, as its computational requirements are half those of the above methods. For MP2, the F12 variant is still the method of choice, as the MP2-F12 and MP2 + CABS + PBE methods have similar costs, but the former provides more accurate results. Additionally, the potential use of an alternative correlation functional instead of a PBE was also examined. Based on the results, we can conclude that replacing the functional is not justified.

For curing the BSIE of CCSD and CCSD(T), we also introduced an alternative, incremental method denoted as CCSD + ΔF12 and CCSD(T)+ΔF12. In this scheme, the total energy is corrected with the CABS (Δ*E*_CABS_) and explicitly correlated MP2 (Δ*E*_F12,c_) contributions. As demonstrated, the new approaches yield surprisingly good results, particularly for reaction and interaction energies. The accuracy obtained closely aligns with those provided by the more expensive CCSD(F12*) and CCSD(F12*)(T+) methods, achieving approximately 1 kcal/mol of error for thermochemical properties even with a double-ζ basis. The costs of the CCSD + ΔF12 and CCSD(T) + ΔF12 approaches are similar to those of the density-corrected CCSD and CCSD(T) methods, respectively, while their accuracy is usually more satisfactory than that for the latter approaches.

The examined corrections can be arbitrarily extended to higher-order CC methods, in particular to those considering full triple and quadruple excitations. Research conducted in this direction will be presented in a subsequent publication.

## Supporting Information Available

Calculated total, correlation, atomization, reaction, and interaction energies (ZIP)
